# Accelerometry reveals limits to use of an energy‐saving anthropogenic food source by a threatened species: A case of Carnaby's cockatoos (*Zanda latirostris*) and canola

**DOI:** 10.1002/ece3.10598

**Published:** 2023-10-09

**Authors:** Karen J. Riley, Kristin Warren, Nicola Armstrong, Lian Yeap, Rick Dawson, Peter R. Mawson, Denis A. Saunders, Christine E. Cooper, Jill M. Shephard

**Affiliations:** ^1^ Centre for Terrestrial Ecosystem Science and Sustainability, Harry Butler Institute Murdoch University Murdoch Western Australia Australia; ^2^ Conservation Medicine Program, School of Veterinary Medicine Murdoch University Murdoch Western Australia Australia; ^3^ Centre for Sustainable Aquatic Ecosystems, Harry Butler Institute Murdoch University Murdoch Western Australia Australia; ^4^ Department of Mathematics and Statistics Curtin University Bentley Western Australia Australia; ^5^ Independent Researcher Waikiki Western Australia Australia; ^6^ Department of Biodiversity, Conservation and Attractions South Perth Western Australia Australia; ^7^ Independent Researcher Weetangera Australian Capital Territory Australia; ^8^ School of Molecular and Life Sciences Curtin University Bentley Western Australia Australia; ^9^ Department of Biological Sciences Macquarie University Maquarie Park New South Wales Australia

**Keywords:** accelerometer, anthropogenic resources, conservation behaviour, energetics, movement ecology, overall dynamic body acceleration

## Abstract

The use of anthropogenic resources is becoming increasingly common as species adapt to human‐induced environmental changes, but their use can expose species to new risks. Understanding how animals exploit these resources is important for guiding conservation management, particularly where species are threatened. The introduction of canola cropping to breeding areas of endangered Carnaby's cockatoo (*Zanda latirostris*) has been attributed to an increase in the birds' reproductive success; however, the seed may be protein‐limiting for nestling growth and its use by cockatoos has been implicated in the emergence of a new disease. We used high‐resolution accelerometer‐capable GPS tags to track eight birds. Accelerometer data were used to calculate overall dynamic body acceleration (ODBA), a proxy for energy expenditure, and to identify and quantify canola and native vegetation foraging behaviours. We used linear mixed models to determine which factors affected patterns of resource use and to determine whether, and to what extent, canola use was associated with reduced energetic and movement costs. We then compared the energetic content of canola seed and native food sources to inform patterns of behaviour and habitat use revealed by our tracking data. Use of canola was associated with reduced movement costs and energy expenditure. However, there was an apparent reluctance to increase foraging on canola above a threshold of time, even when conditions reduced time available to utilise native food sources. While anthropogenic resources may appear to improve population trends in some cases, careful investigations of patterns of resource use are necessary to guide appropriate conservation management efforts. For Carnaby's cockatoos, conservation efforts should focus on retention, protection and expansion of native food sources.

## Graphical abstract

We used accelerometer‐capable GPS tracking to investigate the use of a novel food source by endangered Carnaby's cockatoos (*Zanda latirostris*) during nestling provisioning. We found the importance of canola relative to native food sources increased with increased maximum daily temperature; however, there was a threshold for time spent in canola, even when conditions required time spent foraging on native food sources be reduced. Our findings highlight the care that conservation managers should take to investigate patterns of novel resource use, even where they appear to improve conservation outcomes for threatened species.

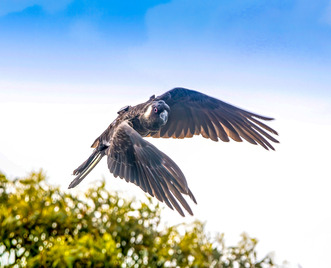

## 1 INTRODUCTION

The use of anthropogenic resources by wild animal populations is becoming increasingly common as species adapt to the rapid and ongoing environmental changes brought about by human activity (Valentine et al., 2020; Vitousek et al., 1997). Human food production, waste disposal and fisheries industries are among a range of anthropogenic activities that provide animals, either advertently or inadvertently, with food sources of high spatio‐temporal predictability (Oro et al., 2013). Such resources, termed predictable anthropogenic food subsidies (PAFS: Oro et al., 2013), are often easier to access than natural resources, resulting in reduced foraging times or energy expenditure (Cama et al., 2012; Grémillet et al., 2008; Oro et al., 2004) and may buffer against the stochasticity inherent in natural food supplies (Bartumeus et al., 2010; Lopez‐Lopez et al., 2013). Use of PAFS can have benefits at the individual or species level including improved body condition (Auman et al., 2008; Rodriguez‐Hidalgo et al., 2010), reproductive success (Kilpi & Öst, 1998; Pérez‐González et al., 2010), individual survival (López‐Bao et al., 2008; McIntyre et al., 2006) and population dynamics (Fuller et al., 2008; Gangoso et al., 2013). Use of PAFS may also have negative impacts, for example, through increased disease or parasite transmission (Carrete et al., 2009; Murray et al., 2015), reduction in food quality or variability (Grémillet et al., 2008; Suzuki, 2017) or through the generation of dependence where supply is unreliable (Singer & Parmesan, 2018). Where these risks translate into reductions in overall fitness, they may become evolutionary traps. These occur when there is a decoupling between environmental cues and the fitness value of a habitat or resource, causing animals to preferentially utilise resources with a lower fitness reward (Robertson et al., 2013; Schlaepfer et al., 2002). Such risks become most problematic where they impact threatened species and may result in conflicting human economic and biodiversity conservation objectives (Valentine et al., 2020). Understanding how threatened species exploit anthropogenic resources may help to guide conservation planning and contribute to the preservation of threatened species.

**FIGURE 1:**
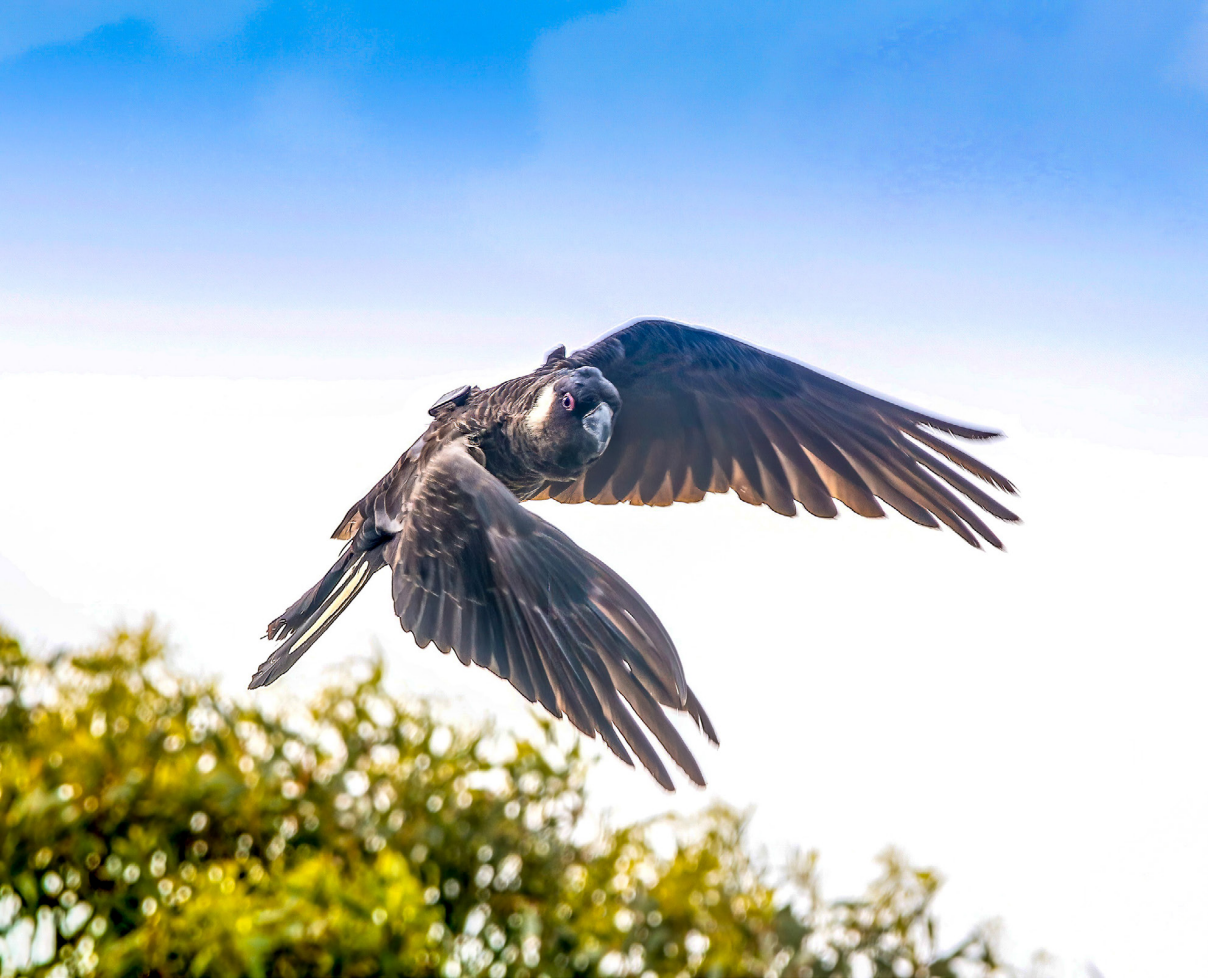
Male Carnaby's cockatoo (*Zanda latirostris*) with back‐mounted GPS tag.

Carnaby's cockatoo *Zanda latirostris* Carnaby, 1948 (Figure 1) is an endangered parrot (Birdlife International, 2022) endemic to the south‐west of Western Australia (WA). Breeding Carnaby's cockatoo have recently begun utilising an anthropogenic food source with apparent benefits to reproductive outcomes (Saunders et al., 2019). Since the beginning of the last century, vast areas of Carnaby's cockatoo breeding habitat have been cleared for agriculture, contributing to a significant contraction in the species’ range and abundance (Saunders, 1990; Saunders & Ingram, 1995). However, the recent adoption of canola (Brassica sp.) to the diet of cockatoos in breeding areas where the seed is a widely grown agricultural crop (Jackson, 2009; Saunders et al., 2014) has been associated with an increase in the success of those breeding populations. A longitudinal study of 10 breeding sites between 1969 and 2013 found nesting success and nestling health correlated with the percentage of intact native vegetation within a 6 and 12 km radius of breeding hollows, respectively (Saunders et al., 2014). A subsequent study found this relationship was no longer apparent after the introduction of canola (*Brassica napus*) to those areas (Saunders et al., 2019).

While it seems canola is offsetting a scarcity of natural food sources for breeding populations, its use is not without potential risk. Nestling growth requires a diet high in protein for the building of body tissue (O'Connor, 1984) and several bird species feed their nestlings selectively on high‐protein foods (Markman et al., 1999; O'Connor, 1984). While the specific dietary protein requirements of Carnaby's cockatoo nestlings are unknown, cockatiel (*Nymphicus hollandicus*) nestlings require a minimum of 20% dietary protein (Roudybush & Grau, 1986). Since protein requirements increase with body size for granivorous birds (Klasing, 1998), requirements for nestling Carnaby's cockatoos, roughly seven times larger than cockatiels, are expected to be somewhat greater than 20%. Canola seed has a protein content of 19.1% compared to the 52%–77% seed protein content of eight species of *Hakea* and *Banksia* (Stock et al., 2013), two plant genera forming the bulk of Carnaby's cockatoos natural diet in breeding areas (Saunders, 1980). We might therefore suspect a diet high in canola seed to be protein‐limiting for the adequate support of Carnaby's cockatoo nestling growth. Furthermore, ingestion of canola sprayed with organophosphates has been implicated in the appearance of a new disease known as Carnaby's cockatoo hindlimb paralysis syndrome, which is emerging as a significant new threat to Carnaby's cockatoo populations (Le Souëf et al., 2020).

In this study, we used high‐resolution accelerometer‐capable GPS tags to investigate the use of canola and native foraging habitats by eight individuals of Carnaby's cockatoo during provisioning of nestlings. We aimed to determine which external factors affected patterns of canola and native foraging habitat use and to determine whether, and to what extent, canola use was associated with reduced energetic and movement costs. Optimal foraging theory suggests that animals adopt foraging strategies that maximise energy intake while minimising expenditure of energy and time (Ydenberg et al., 1994). Energetic costs associated with movement can be approximated using overall dynamic body acceleration (ODBA), calculated as the sum of acceleration recorded along three spatial planes (Wilson et al., 2006). We used ODBA as a proxy for energy expenditure, along with measurements of distance travelled and time spent at rest, to assess whether patterns of habitat use impacted the cost of provisioning in Carnaby's cockatoos. Flight is an energetically expensive activity (Norberg, 1996), and time spent engaged in flight has a positive correlation with energy expenditure (Nudds & Bryant, 2000). We therefore predicted that the use of canola, which is often available closer to nesting hollows than native foraging patches, would be associated with reduced provisioning costs. Second, we examined which external factors influenced the time and energy birds expended in canola and native foraging habitats. Since Carnaby's cockatoos use rest as a thermoregulatory strategy (Saunders, 1977), we predicted that days with higher maximum temperatures would be associated with more time spent at rest and therefore more time foraging on the more proximal canola foraging resource. Finally, we aimed to compare the energetic content of canola seed and native food sources to further inform the patterns of behaviour and habitat use revealed by our tracking data.

## 2 MATERIALS AND METHODS

**FIGURE 2:**
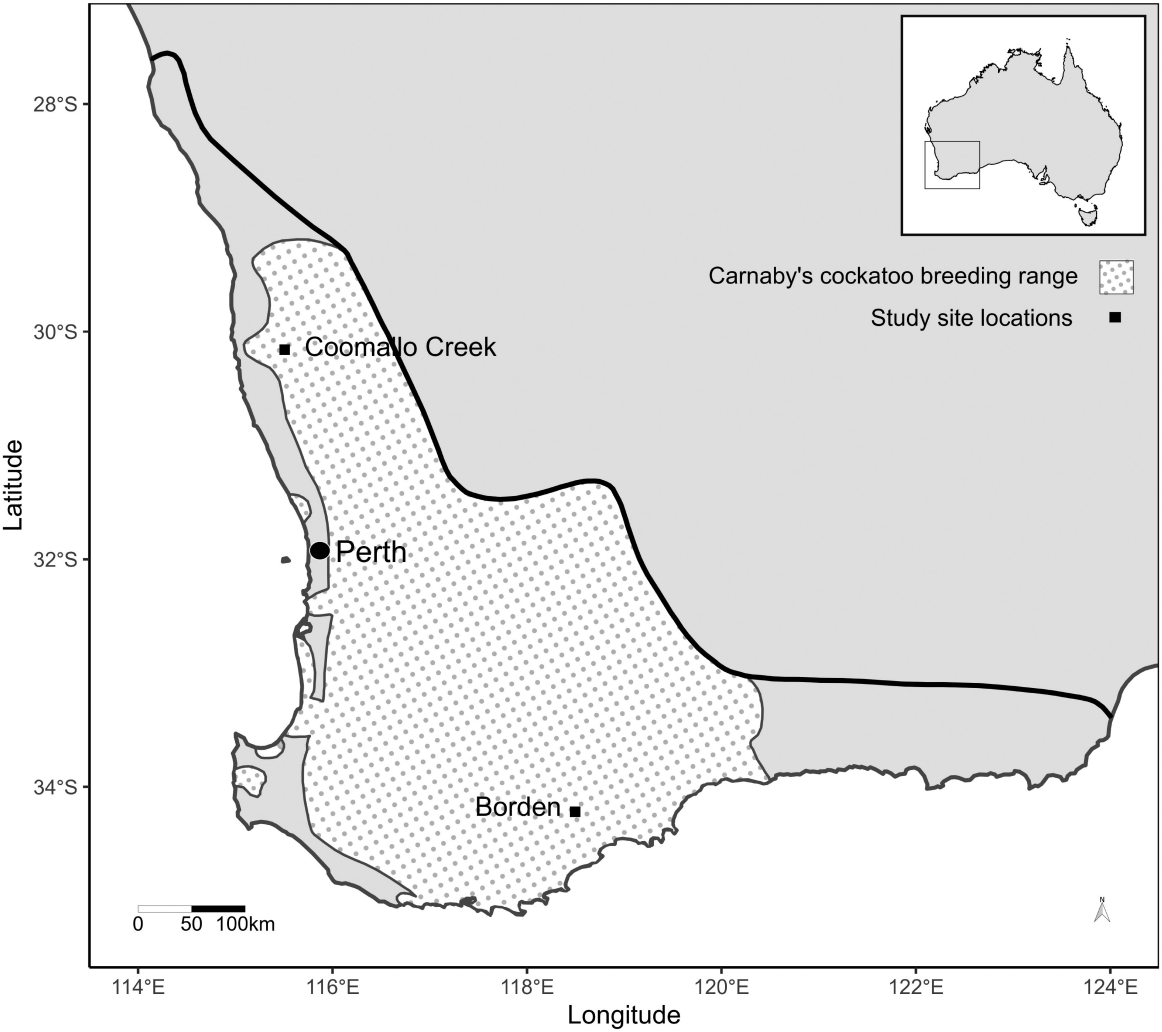
Study site locations in relation to Carnaby's cockatoo (*Zanda latirostris*) nonbreeding range (south of solid line) and breeding range [dotted area; adapted from Department of Environment and Conservation (2012)].

Fourteen Carnaby's cockatoos were equipped with trackers, of which eight yielded data for use in our analyses; six tags were destroyed by the birds prior to generating data. Tracking took place in two breeding seasons (2017 and 2018) with each bird tracked in one season only (Table S1). The tracked individuals nested inside private farming property in the northern and southern areas of the WA's wheatbelt region; Coomallo Creek (200 km north of Perth; 30°9′3″S, 115°30′30″E) and Borden (350 km south‐east of Perth; 34°13′10″S, 118°29′50″E) (Figure 2). Birds nested in patches of wandoo (*Eucalyptus wandoo*) where artificial hollows had been installed to supplement the availability of the natural hollows in which Carnaby's cockatoos breed (Saunders, 1982; Saunders et al., 2020). Breeding areas were surrounded by cleared agricultural land and patches of uncleared proteaceous shrubland, which provide the traditional food source for breeding cockatoos (Saunders, 1980). The percentage of uncleared native vegetation within 12 km of each site was approximately 25% at Coomallo and 17% at Borden (Saunders et al., 2019). Around 80 and 20 nestlings per season are produced at the Coomallo Creek and Borden sites, respectively (Saunders et al., 2019).

Study birds were adults provisioning nestlings at least 3 weeks old at the time of tag deployment; tagging birds with younger nestlings was avoided to reduce the potential risk of nest abandonment. Nestlings were aged by measuring the folded left wing and comparing the measurement to a logistical curve describing the increase in wing length with age (Saunders, 1982; Saunders et al., 2015). Adult birds were trapped in the evening as they entered the hollow to provision nestlings. The first bird to enter the hollow was trapped, regardless of sex, which is readily determined by sexually dimorphic differences in beak, eye‐ring and cheek patch colour. Due to chance in trapping, only males were tagged at Borden and only females were tagged at Coomallo Creek. The trapped bird was removed from the hollow and transported to a field clinic for tag attachment.

### 2.1 Tags and tag attachment procedures

Study birds were fitted with both a Platform Transmitter Terminal Argos satellite tag and a solar GPS tag incorporating a 20 Hz tri‐axial accelerometer. GPS tags collected high‐resolution location data (±20 m) and accelerometer data for fine‐scale movement and energetic analysis. Argos tags enabled on‐the‐ground tracking for the observation of foraging activity. Tags were attached under isoflurane inhalation anaesthesia to minimise stress.

Argos tags (17 g; Telonics TAV‐2617) were attached to the ventral side of the two central tail feathers using braided fishing line. To attach GPS tags [7.5 g; University of Amsterdam Bird Tracking System (UvA‐BiTS) 2CDSe], a plastic backing plate was first attached to four feathers, 10 mm below the shoulder joints using adhesive cloth tape. The tag was then secured to the backing plate using glue and braided fishing line [see Yeap et al. (2017) for detailed tag attachment methods]. This attachment method was stable with no visible movement in tags during flight. The combined weight of tags was 3.7% of mean adult body mass, conforming to ethical standards (Kenward, 2001). Tags were moulted out with the feathers to which they were attached, unless they were removed by the birds earlier during preening or allopreening. Nests were monitored in the days following tagging to ensure tag attachment procedures did not prevent parent birds from provisioning nestlings.

Birds were fitted with an Australian Bird and Bat Banding Scheme (ABBBS) numbered stainless steel band on the right leg and a unique combination of two‐coloured bands on the left leg (ABBBS permit number 1862) to facilitate identification in the field. At the conclusion of procedures, female birds were returned to the hollow until first light when trap lids were removed. Males, which do not usually brood nestlings overnight, were released on the ground close to the hollow following recovery from anaesthetic.

This study was conducted with all appropriate approvals and permits: WA Department of Biodiversity, Conservation, and Attractions (DBCA) Animal Ethics Committee project approval (#2014–23 and #2017–21); Scientific Licenses SC001289 and SC001405; and Murdoch University Animal Ethics Permit RW2828/16.

### 2.2 Data collection and retrieval

Daily maximum temperatures were obtained from the Australian Government's Bureau of Meteorology (www.bom.gov.au/climate/data/) from the closest weather station to each site (distances: Coomallo Creek—26 km; Borden—28 km).

GPS data were downloaded remotely to a base station via the tag's Zigbee wireless transceiver. Several relay antennae were positioned throughout the breeding area, with data downloads commencing whenever tagged birds entered the antennae's field of reception. Data were uploaded from the base station to the UvA‐BiTS e‐infrastructure platform, with postprocessed data accessed via the UvA‐BiTS Virtual Lab portal (www.UvABiTS.nl/virtual‐lab; Bouten et al., 2013).

GPS tags were programmed to collect data at 2.5–15‐min intervals between 05:00 and 18:30 depending on solar recharge and every 30 min at night. The mean interval of sampled data for all tags was 6.27 ± 10.19 mins (max interval = 15 h 9 mins). Sixty‐six per cent of sampled daytime data had a duration of 2.5 min. Tri‐axial acceleration was measured at 20 Hz for 1 s with each GPS location. Argos tags were programmed to communicate fortnightly for 4 h (06:00–10:00); during this time, birds were located using an Argos AL‐1 PTT Locator (Communications Specialists) and observed for foraging activity.

### 2.3 Data processing and analyses

Only data collected during the period of nestling provisioning were analysed in this study.

Separate linear mixed models (LMMs) were constructed to determine which factors affected three daily metrics of bird activity: mean OBDA, distance travelled and time spent at rest. Since foraging in canola and native vegetation requires different foraging strategies, separate LMMs were also constructed to determine which factors affected time and energy spent foraging in each habitat (Table S2). Explanatory variables tested included site, daily maximum temperature, daylength, nestling age, number of nestlings and number of provisioning hollow visits (PHVs).

Due to the uneven interval in sampled data, data were resampled to a minimum interval of 10 mins to ensure all periods of activity were evenly sampled. Days with any interval greater than 60 mins were excluded from LMMs. Eighty per cent of daytime data in the resampled data set had an interval between 10 and 15 mins (mean = 14.25 ± 5.6 mins).

Relative energy expenditure corresponding to each GPS location was approximated using accelerometer data through calculation of ODBA (ms^−1^). Overall dynamic body acceleration (ODBA) has elsewhere been correlated with more direct measurements of energy expenditure such as doubly labelled water and oxygen consumption (Gleiss et al., 2010; Halsey et al., 2009; Wilson et al., 2006). Following methods described by Wilson et al. (2006), we subtracted the static component of acceleration (i.e. acceleration due to Earth's gravitational field) from measurements within each of the three movement axes: *x* (surge), *y* (sway) and *z* (heave). Static acceleration was approximated as the mean acceleration along each axis per 1‐s frame, which was considered a suitable alternative to the running mean used by Wilson et al. (2006) given the 1‐s frame length in this study (A. Gleiss, personal communication, 2018). The resulting measurements were converted to absolute positive units and summed across all three axes to determine overall dynamic acceleration, or acceleration due to body movement.

Distances between consecutive GPS locations were calculated using the ‘fossil’ package (Vavrek, 2011). Data were coded to day (between civil dawn and civil dusk) or night using the ‘maptools’ package (Bivand & Lewin‐Koh, 2019).

A behavioural classification of either ‘resting’, ‘foraging’ or ‘flying’ was assigned to each GPS location by processing the corresponding accelerometer data frame through an automated classification model developed for black cockatoos by Yeap et al. (2021). Development of the classification model involved manual classification of bird behaviours by an expert observer, which was then used to train a machine‐learning algorithm to recognise distinctive accelerometer signatures produced during each behaviour. The model's algorithm was able to identify these behaviours with 86% accuracy (Yeap et al., 2021; see Supporting Information for validity testing of classifications applied to data in this study). Daytime hours spent at rest were calculated by summing the duration between subsequent GPS locations following each location classified as resting.

To identify instances of foraging on canola, polygons were drawn in Google Earth (2022, Version 7.3.6.9345, accessed 03/05/2021) around areas of canola cropping or canola grain spills and extracted as .kml files for further analysis. GPS locations of the behavioural class ‘foraging’ intersecting with these polygons were extracted using the ‘sp’ package (Bivand et al., 2013); these GPS locations were classified as foraging on canola.

Foraging on native vegetation was classified as all locations with the behavioural class ‘foraging’ occurring outside canola crop polygons. During the development of the behavioural classification algorithm by Yeap et al. (2021), unspecified behaviours including preening and aggressive interactions (which accounted for <4% of total observations) were incorporated into the ‘foraging’ behavioural class. Behaviours associated with breeding such as entering and exiting the hollow and feeding the nestling were not incorporated into the classification algorithm, which was developed using observations of nonbreeding birds. It was expected that these behaviours would be captured as ‘foraging’ by the classifier (L. Yeap, pers. comm., 2018). To avoid overclassification of foraging on native vegetation by the inclusion of breeding behaviours, as well as preening and aggressive social interactions which were most commonly observed in the breeding area, a 750 m buffer around nesting hollows was applied when classifying foraging on native vegetation. We deemed this buffer appropriate as our observations revealed very little foraging occurred within the wandoo woodland nesting habitat; foraging on native vegetation occurred almost exclusively within proteaceous shrubland several kilometres or more from nesting hollows. A small amount of foraging on exotic pine (*Pinus pinaster*) occurred at the Borden site only. These were incorporated into the foraging on native vegetation classification. Their inclusion was deemed inconsequential as they accounted for <1% of all foraging locations. Foraging within a small patch of wild radish (*Raphanus raphanistrum*); a competitive and prolific agricultural weed from the same plant family as canola (Blackshaw et al., 2002) was identified at the Coomallo Creek site; this activity was incorporated into the canola foraging classification. Time (hours) spent foraging within each habitat was calculated by summing the duration between subsequent GPS locations following each location classified as foraging.

Daytime hollow visits were counted using the R ‘recurse’ package (Bracis et al., 2018) with hollow revisit thresholds set to 30 m and 10 mins. The distance threshold accounted for error in GPS location data and to capture hollow visits by provisioning males which do not necessarily always enter the hollow, but may instead feed the female close to the hollow (Saunders, 1982). Provisioning hollow visits (PHVs) were classified as any hollow visit preceded by a trip to a foraging area. Recurse analysis failed to detect an evening hollow visit on 22% of days due to GPS tag data collection scheduling dropping to 30‐min intervals at 18:30, at least 47 min prior to sunset during the study period. Saunders (1982) and our own observations revealed a provisioning visit was always made in the evening following afternoon foraging and prior to roosting. These visits were often brief, lasting as little as several minutes, and could easily have been missed with a data collection interval of 30 min. In these instances, one PHV was added to the day's count.

Nestlings were assumed to have fledged 76 days after hatching (Saunders et al., 2015) unless fledge dates were known to differ from this based on observations at the hollow or GPS movements. Data from Day 1 post‐tag deployment were excluded from analyses. Females usually raise one nestling from two eggs laid (Saunders, 1982). For nests with two nestlings, nestling age was assigned according to the youngest nestling.

### 2.4 Statistical data analyses

After data resampling, 123 bird‐days (2–29 days per bird) were available for analyses in LMMs (Table S1).

Metrics used in LMMs were measured over an individual bird‐day with the statistic for each bird on each day considered a single data point (Table S2). All models included random intercepts through the incorporation of individual as a random effect. For models describing factors affecting the daily activity metrics of mean ODBA, distance travelled and time at rest, explanatory variables tested included the proportion of foraging time spent in canola (calculated by dividing the number of canola foraging locations by all foraging locations), site, daily maximum temperature, PHVs, daylength, nestling age and number of nestlings. For models describing factors affecting time and energy expended during foraging, daily hours spent foraging and mean daytime ODBA of foraging locations within each habitat were analysed as the response variables in separate LMMs. Explanatory variables included site, maximum temperature, PHVs, daylength, nestling age and number of nestlings.

Linear mixed models (LMMs) were constructed using the ‘lme4’ package (Bates et al., 2015). After checking for collinearity of explanatory variables (all Pearson's correlation coefficients were < 0.25), we began with a full model including all variables and removed nonsignificant variables having the highest *p*‐value in a backward stepwise fashion. After each removal, Akaike's Information Criterion (AIC) was compared between the full model and the reduced model. The most parsimonious final model was selected, having the fewest explanatory variables and the lowest AIC. Residuals were inspected using QQPlots and Pearson's plots to ensure model assumptions of homogeneity and normality were met (Schielzeth et al., 2020; Zuur et al., 2009). Outliers were tested to ensure they did not influence final model outcomes (see Supporting Information). Statistics for each final model were generated using the Anova() function (type = ‘II’). Estimated marginal means or coefficients were calculated using the ‘emmeans’ package (Lenth, 2020). Marginal effects were plotted using the ‘sjPlot’ package (Ludecke, 2020). Values are presented as mean ± standard deviation unless stated otherwise.

All analyses were conducted in R Core Team (2019).

### 2.5 Energetic analysis of canola and native plant foods

Samples of foraging items, identified during direct observation of foraging activity or by feed residues discovered during retrospective investigation of GPS tracks, were collected from across the foraging range at both sites. Throughout the study, feeding was recorded on eight banksia, six hakea and one eucalypt species. Exotic species included canola, pine and wild radish.

Sufficient samples for bomb calorimetry were collected from six banksia and four hakea species, with at least four banksia and two hakea species collected at each site. Seeds were extracted from the cones or fruits, weighed using a Sartorius analytical balance (BCE224I‐1S; Sartorius Lab Instruments) and dried over several days at 50°C to a constant weight. Dried seeds were ground to a powder before being pressed into pellets of around 1 g and embedded with a cotton ignition thread. Pellets were redried and weighed before undergoing measurement for gross calorific value using an oxygen bomb calorimeter (Ika™ C2000) and calibrated using a benzoic acid standard.

## 3 RESULTS

### 3.1 General foraging activity

Foraging on canola and native food sources took place within a range of 7.81 and 13.54 km of nest hollows, respectively (Figure S1; see Table 1 for foraging statistics).

**TABLE 1:** General foraging statistics (±SD) for Carnaby's cockatoos (*Zanda latirostris*) tracked at Borden and Coomallo Creek.

	Borden	Coomallo Creek	Study
*Distance travelled from nest*			
Maximum (km)	13.55	11.11	13.55
Mean daily maximum (km)	5.98 ± 0.24	6.40 ± 0.28	6.18 ± 0.18
*Canola foraging (n = 2661 locations)*			
Mean distance from nest (km)	2.94 ± 0.04	0.97 ± 0.03	2.18 ± 0.03
Daily time spent (h)	1.37 ± 0.58	1.19 ± 0.86	1.28 ± 0.73
Mean ODBA (ms^−1^)	2.53 ± 0.06	2.84 ± 0.08	2.65 ± 0.05
*Native vegetation foraging (n = 8446 locations)*			
Mean distance from nest (km)	4.88 ± 0.03	3.84 ± 0.03	4.34 ± 0.02
Daily time spent (h)	3.78 ± 0.58	4.16 ± 0.86	3.96 ± 1.57
Mean ODBA (ms^−1^)	3.70 ± 0.04	3.72 ± 0.04	3.71 ± 0.03

Birds foraged during two main periods of the day, between 06:00 and 09:00 and between 15:00 and 18:00 (Figure S2a), separated by a period of roosting. Birds provisioned nestlings a minimum of two and a maximum of five times per day, with an overall average of 2.59 ± 0.07 daily PHVs (*n* = 291). Additional (*n* > 2) visits were made on 46% of days and occurred in two peaks; one between 08:00 and 09:00 and another between 13:00 and 15:00 (Figure S2b).

### 3.2 Effect of foraging on canola and other external factors on energetic and movement metrics

**FIGURE 3:**
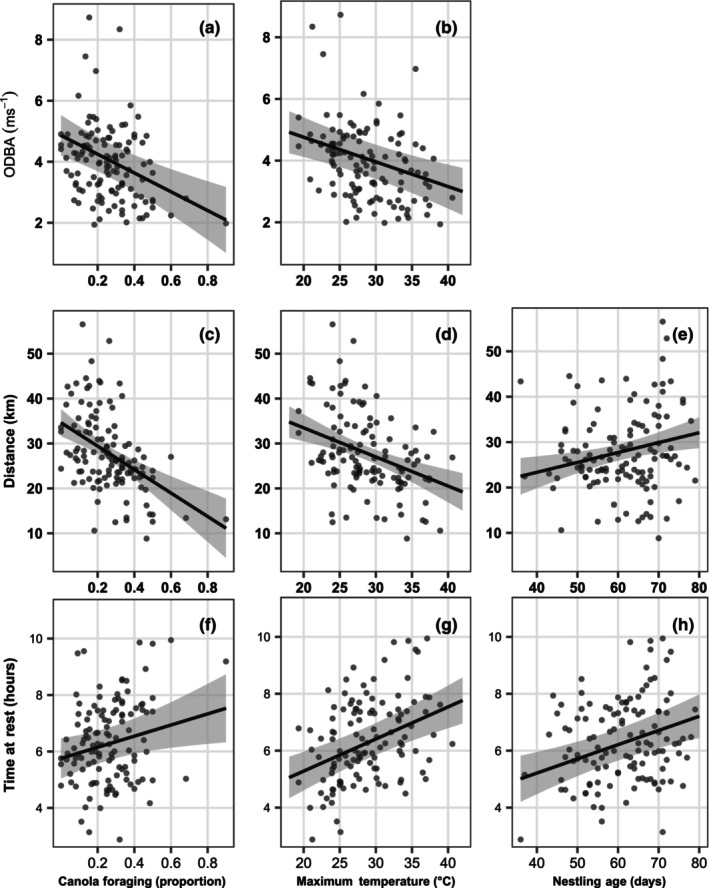
Marginal effect plots for fixed effects significantly affecting Carnaby's cockatoo (*Zanda latirostris*) activity metrics. Each row of plots represents marginal effects for a separate linear mixed model (LMM); the response variables in the models are mean daytime overall dynamic body acceleration (ODBA; ms^−1^) (a, b), daily distance travelled (km) (c, e) and time spent at rest (h) (f–h). Each point on the plot represents an individual bird‐day, and grey lines represent 95% confidence intervals.

Increased time foraging on canola led to a significant reduction in all three activity metrics (Figure 3 and Table S3). As the proportion of foraging on canola increased, birds had significantly reduced mean daytime ODBA (Figure 3a; χ^2^ = 17.87_1_, *p* = 2.36 × 10^−5^) and daily distance travelled (Figure 3c; χ^2^ = 26.50_1_, *p* = 1.71 × 10^−6^) and spent significantly more time at rest (Figure 3f; χ^2^ = 5.68_1_, *p* = .01). Similarly, as maximum daily temperature increased, birds had significantly reduced mean daytime ODBA (Figure 3b; χ^2^ = 16.72_1_, *p* = 4.33 × 10^−5^) and daily distance travelled (Figure 3d; χ^2^ = 19.17_1_, *p* = 2.73 × 10^−5^) and spent significantly more time at rest (Figure 3g; χ^2^ = 25.41_1_, *p* = 4.62 × 10^−7^).

As nestlings aged, birds travelled further (Figure 3e; χ^2^ = 6.96_1_, *p* = .01) and spent significantly more time at rest per day (Figure 3h; χ^2^ = 13.18_1_, *p* = 1.23 × 10^−4^). Overall, there was no effect of nestling age on mean daytime ODBA.

### 3.3 Factors affecting time and effort allocated to foraging on canola and native vegetation

**FIGURE 4:**
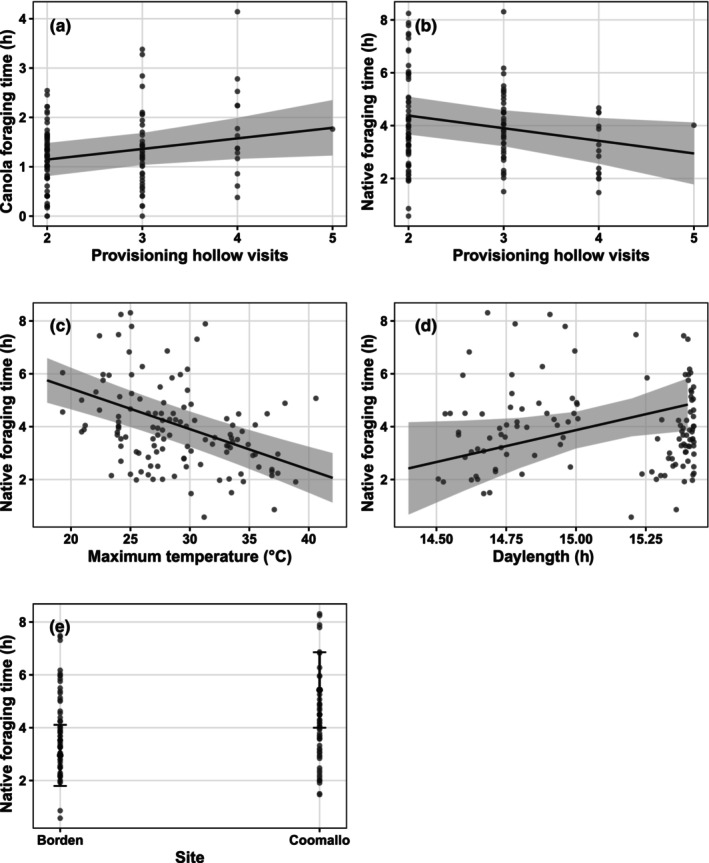
Marginal effects plots for fixed effects significantly affecting the number of hours spent foraging by Carnaby's cockatoo (*Zanda latirostris*) in canola (a) and native vegetation (b–e) in the Western Australian wheatbelt. Each point on the plot represents an individual bird‐day. Grey shading represents 95% confidence intervals. Note in plot (e); Borden and Coomallo Creek data were sampled from all males and females, respectively.

A greater number of PHVs was associated with more time foraging on canola (Figure 4a; χ^2^ = 4.79_1_, *p* = .03) and less time foraging on native vegetation (Figure 4b; χ^2^ = 5.29_1_, *p* = .02). Hotter days (Figure 4c; χ^2^ = 38.15_1_, *p* = 6.55 × 10^−10^) were also associated with less time foraging on native vegetation. Conversely, cockatoos spent more hours foraging in native vegetation during periods of longer daylength (Figure 4d; χ^2^ = 4.12_1_, *p* = .04). A significant effect of site was also found, with birds from Coomallo Creek spending more foraging hours in native vegetation compared with those from Borden (Figure 4e; χ^2^ = 4.90_1_, *p* = .02).

**FIGURE 5:**
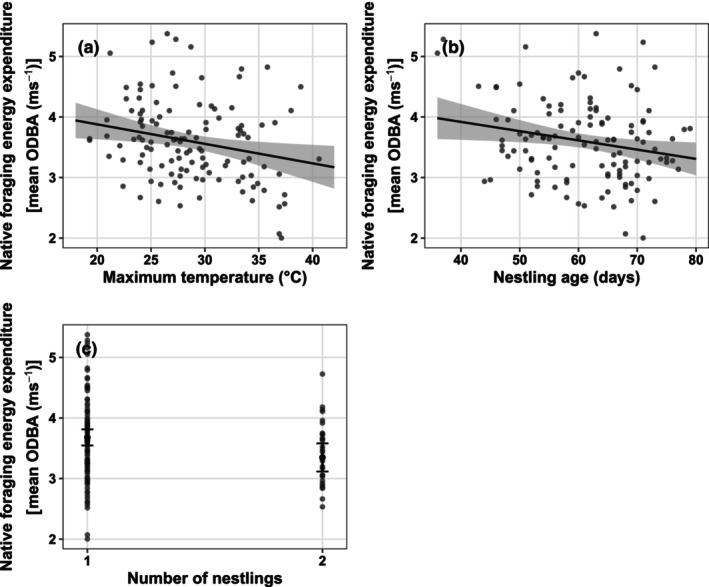
Marginal effects plots for fixed effects significantly affecting Carnaby's cockatoo (*Zanda latirostris*) energy expended while foraging within native vegetation. Native foraging energy expenditure calculated as mean daily overall dynamic body acceleration (ODBA; ms^−1^) of all locations classified as foraging within native vegetation. Each point on the plot represents an individual bird‐day. Grey shading represents 95% confidence intervals.

Energy spent foraging within canola was not affected by any of the fixed effects tested in the model. However, birds decreased energy expended foraging in native vegetation as maximum daytime temperatures increased (Figure 5a; χ^2^ = 5.68_1_, *p* = .02) and as nestlings aged (Figure 5b; χ^2^ = 6.47_1_, *p* = .01). Birds with two nestlings also expended significantly less energy foraging within native vegetation than those with one nestling (Figure 5c; χ^2^ = 5.29_1_, *p* = .02). See Table S4 for models.

### 3.4 Energy content of food items from canola and native foraging habitats

Banksia and hakea made up the majority of native vegetation foraging observed across sites and contained around 25% fewer calories per mass of seed (mean = 21.42 kJg^−1^, seed mass range = 0.01–0.12 g) than canola (28.7 kJg^−1^, mean seed mass = 0.0035 ± 0.0007 g; Table S5). The energy content of pine and wild radish was similar to that of canola at 26.3 kJg^−1^ (mean seed mass = 0.01 ± 0.006 g) and 27.5 kJg^−1^ (mean seed mass = 0.003 ± 0.001 g), respectively.

## 4 DISCUSSION

For Carnaby's cockatoos provisioning nestlings, days where birds allocated a greater proportion of foraging time to canola were associated with reduced average ODBA, or overall energy expenditure (Figure 3a). With flapping flight incurring high energetic costs per unit time (Norberg, 1996), the fact that canola foraging activity took place an average of 60% closer to nesting hollows than foraging in natural vegetation patches likely contributed in large part to the energy savings afforded by canola foraging. By increasing the proportion of foraging time spent in canola from 20% to 40%, birds were able to reduce their overall distance travelled by around 5 km per day (Figure 3c), an almost 17% reduction in travel costs. The foraging strategy required when utilising canola was also less energy intensive, as indicated by the 30% lower ODBA of canola foraging locations compared with native vegetation foraging. Foraging in canola requires picking the scattered seed from the ground or, in the case of concentrated spills, scooping the seed using the lower mandible. In native habitat, birds must employ a more energetically demanding arboreal foraging strategy and are required to open woody cones to access native nuts and seeds.

Higher daily maximum temperatures resulted in significantly reduced distance travelled and overall energy expenditure, as well as increased time at rest (Figure 3b,d,g), consistent with a reduction in activity as a thermoregulatory tool for birds occupying hot environments (Davies, 1982). Saunders (1977) reported this behaviour for Carnaby's cockatoos, noting birds became inactive and retreated to deep shaded branches during the heat of the day. Our data lend further support to this, with birds spending roughly 30 min additional time at rest per day for every 5°C increase in daily maximum temperature (Figure 3g), as well as a concurrent decrease in distance travelled (Figure 3d) and overall energy expenditure (Figure 3b). Reducing total foraging time on hot days, as well concentrating foraging activities to the cooler parts of each day (Figure S2a), reduces metabolic heat production when heat dissipation is most problematic.

Due to decreased time available for foraging under hot conditions, we expected higher temperatures to be associated with increased time or energy spent foraging on canola. Our data found no evidence to support this; birds dedicated around 1 h 30 mins each day to foraging on canola and expended similar energy doing so, regardless of temperature, number of nestlings, nestling age or daylength. Instead, birds significantly reduced time and energy spent foraging on native vegetation in response to high temperatures (Figures 4c and 5a). Birds spent around 4 h each day foraging in native vegetation, which decreased by roughly 30 min with each 5°C increase in temperature (Figure 3c). The reduction in foraging energy expenditure suggests a concurrent decrease in the intensity of foraging activity as temperatures increased. These findings lend support to the work of Saunders (1982) who noted a significant negative correlation between nestling daily weight gain and daily maximum temperature in a declining breeding population in the wheatbelt prior to the introduction of canola.

The apparent reluctance of provisioning birds to increase foraging in canola relative to native vegetation may result from attempts to balance the intake of high‐energy canola with high‐protein native seed. Optimal foraging theory suggests that an animal should adopt the foraging strategy that provides the most energetic gain for the least energetic output (Ydenberg et al., 1994). Comparing the overall rate of energy intake in each habitat would have required quantifying the rate of ingestion as well as assimilation efficiency for each seed type; this is difficult to determine in a field setting and was beyond the scope of this study. However, despite the small size of canola seeds (Table S5) we might assume that canola provides the more energy‐efficient foraging strategy given its reduced travel and handling costs, opportunity for bulk feeding and the higher energy content per mass of seed identified through our calorimetric analysis. While early optimal foraging studies assumed energy to be the only currency at play in shaping foraging strategies, more recent studies have demonstrated the ability of some species to balance specific dietary nutrients by controlling the proportional intake of food types in the diet (Jensen et al., 2012; Simpson et al., 2004; Thompson et al., 1987). An apparent recognition in provisioning Carnaby's cockatoo of the need to limit canola intake supports the assumption that the grain may be limiting in its ability to support growing nestlings. This also provides evidence against canola in our study sites acting as a potential ecological trap, which occurs when animals preferentially utilise resources with a lower fitness reward (Robertson et al., 2013; Schlaepfer et al., 2002). Habitat preference, which is often assessed by relating the use of habitat to its availability (Manly et al., 2007; Thomas & Taylor, 1990), appears to be for native habitats for birds in this study. Despite native patches occupying less space in the landscape and occurring at greater distances from nest hollows, birds' time investment was three times greater in native habitats compared with canola.

Provisioning effort decreased across the nestling period with birds spending significantly more time at rest and less time and energy foraging on native vegetation as nestlings aged. This is consistent with the common strategy among altricial birds of ‘parental meanness’ (Davies, 1978; Trivers, 1974) whereby birds decrease provisioning effort in the latter part of the nestling period to encourage fledging. Saunders (1982) reported that nestlings at Coomallo Creek gained weight until 53 days, after which time mass plateaued before decreasing just prior to fledging. Nearly 80% of our data were collected after Day 53. The increase in daily distance travelled possibly results from exhaustion of more proximal food sources during the nestling period as well as an increase in exploratory movements close to fledging as birds prepare to depart the breeding area.

The intelligence of parrots (Psittacines) and their strong beaks make them challenging subjects for tracking studies due to increased risk of subjects removing or interfering with tracking devices (Herrod et al., 2014). This study faced additional challenges associated with the bird's endangered status and its hollow dwelling breeding habit. As a result, the study is limited by its small sample size, making it difficult to draw conclusions about the effects of sex, site and number of nestlings, as well as individual variation in foraging strategy. An effect of individual was suggested by significant random intercepts in some of the models (Tables S3 and S4). This may be age‐related with increased reproductive success in several bird species resulting from improvements in foraging efficiency as birds age (Lack, 1966; Saraux & Chiaradia, 2021). This has been demonstrated for Carnaby's cockatoos with older, more experienced birds more likely to attempt to raise two nestlings rather than one (Saunders et al., 2014). However, larger sample sizes with sexes represented across both sites would be needed to confirm the true effects of sex, site and number of nestlings, as well as identifying any individual foraging strategies for provisioning Carnaby's cockatoos.

Given the temperature‐dependent nature of foraging behaviour, there is the potential for canola to act as a buffer against the impacts of climate change, with average temperatures in south‐western Australia predicted to increase by between 1°C and 4°C by 2090 (Department of Water and Environmental Regulations, 2021). While foraging effort expended on canola did not significantly increase with temperature, its relative importance in the diet increased since significantly less foraging time was available for foraging on native food sources. Greater dependence on canola will leave breeding populations more vulnerable to the associated risks, such as exposure to organophosphates and potential dietary deficiencies. Cockatoos nesting at sites with less available native vegetation than those in our study may also demonstrate greater dependence on canola. This could be confirmed by further research expanding our experimental design to sites with a wider range of accessible native vegetation.

Further future research could also attempt to predict changes in population dynamics if canola ceased to be available to breeding Carnaby's cockatoos. The immediate future of canola as an agricultural crop in WA seems secure with a record harvest in 2021 (Australian Oilseeds Federation, 2021). However, adverse effects on canola yield and quality are expected to result from climate change (Namazkar et al., 2016), with predicted yield reductions in the WA wheatbelt over the next 50 years of up to 25% (Vernon & Van Gool, 2006). Studies have predicted a reduction in the population of a number of bird species following the loss of the PAFS on which they depend (Duhem et al., 2008; Oro et al., 2008; Parra & Tallería, 2004). Oro et al. (2013) predict that populations heavily exploiting PAFS may respond to their loss by declining to even smaller population sizes than before appearance of the resource, making forecasting particularly important where the species exploiting the resource is endangered.

This study contributes to the growing body of literature documenting the effect of anthropogenic food sources on wild animals; it also highlights the importance of investigating patterns of use, even where use appears to improve conservation outcomes such as breeding success. In the case of Carnaby's cockatoos, conservation managers should take care to ensure that canola is not seen as a suitable replacement for native vegetation, nor as an offset for further removal of native vegetation in breeding areas.

## AUTHOR CONTRIBUTIONS

**Karen Riley:** Conceptualization (lead); data curation (lead); formal analysis (lead); investigation (lead); methodology (supporting); project administration (lead); writing – original draft (lead). **Jill M. Shephard:** Conceptualization (supporting); formal analysis (supporting); investigation (supporting); methodology (supporting); project administration (supporting); supervision (supporting); writing – review and editing (supporting). **Kristin Warren:** Conceptualization (supporting); investigation (supporting); methodology (supporting); project administration (supporting); supervision (supporting); writing – review and editing (supporting). **Nicola Armstrong:** Formal analysis (supporting); writing – review and editing (supporting). **Lian Yeap:** Methodology (supporting); writing – review and editing (supporting). **Rick Dawson:** Investigation (supporting); methodology (supporting). **Peter R. Mawson:** Investigation (supporting); methodology (supporting). **Denis A. Saunders:** Investigation (supporting); methodology (supporting); writing – review and editing (supporting). **Christine E. Cooper:** Methodology (supporting); writing – review and editing (supporting).

## FUNDING INFORMATION

This project was supported with financial assistance provided by Newmont Boddington Gold and South32; PTI Architecture. Research Grant Number 15853, as well as in‐kind assistance provided by DBCA and Birdlife WA.

## CONFLICT OF INTEREST STATEMENT

None to declare.

## Supporting information


Data S1:

## Data Availability

Due to the threatened status of Carnaby's cockatoo (Birdlife International, 2022) and the risk of nest poaching for the illegal aviculture trade, we are unable to publish our GPS data, which include the location of nest hollows on private property. We would be happy to discuss data sharing upon reasonable request to the corresponding author.
