# Supplementary material for: Accelerometry reveals limits to use of an energy‐saving anthropogenic food source by a threatened species: A case of Carnaby's cockatoos (Zanda latirostris) and canola
Source: Ecol Evol. 2023 Oct 9;13(10):e10598. doi: 10.1002/ece3.10598 (PMC10560869; doi:10.1002/ece3.10598)
Supplement: Supplementary file 1 — Data S1: [file ECE3-13-e10598-s001.pdf]

SUPPORTING INFORMATION

METHODS

Behavioural Classifications

The validity of behavioural classifications generated in this study using the algorithm developed by Yeap *et al.* (2021) was confirmed by calculating mean GPS speeds for data classified to each behaviour (mean GPS speed ± SD: flying = 8.56 ± 5.64m/s^{-1}; foraging = 0.85 ± 1.20 m/s^{-1}; resting = 0.69 ± 0.86 m/s^{-1}). Mean GPS speeds were appropriate for each behaviour.

Exploration of outliers in LMMs

Outliers were investigated through calculation of Cook's distances (*D*) using the '*influence.ME'* package in R (Nieuwenhuis, Te Grotenhuis & Pelzer 2012). Following the suggestion of Bollen and Jackman (1985) that data points with values of *D* above "*n*/4" warrant investigation, data points above this threshold within each model were identified. These data were removed and the model re-run to ensure that the outcome of the model was unaffected.

REFERENCES

Bollen, K.A. & Jackman, R.W. (1985) Regression diagnostics: An expository treatment of outliers and influential cases. *Sociological Methods & Research,* **13,** 510-542.

Nieuwenhuis, R., Te Grotenhuis, M. & Pelzer, B. (2012) influence.ME: Tools for Detecting Influential Data in Mixed Effects Models. *R Journal,* **4,** 38-47.

Yeap, L., Warren, K.S., Bouten, W., Vaughan-Higgins, R., Jackson, B., Riley, K., Rycken, S. & Shephard, J.M. (2021) Application of tri-axial accelerometer data to the interpretation of movement and behaviour of threatened black cockatoos. *Wildlife research,* **49,** 100-110.

Table S1: Summary data for each tag and mean daily statistics used in linear mixed models (LMMs). Mean daily statistics were calculated using daytime data (between civil dawn and civil dusk) re-sampled to 10-minute intervals. Hours spent foraging or at rest were calculated by summing the duration between subsequent GPS locations following each location classified as either foraging or resting

									Mean daily statistics (± SE) used in LMMs						
										Foraging hours spent in:					
**Site**	**Year**	**Bird ID**	**Sex**	**Nestlings**	**UVA Tag ID**	**Track start date**	**Track end date**	**Days in LMM**	**Locations**	**Canola**	**Native vegetation**	**PHVs**	**Distance (km)**	**ODBA (m/s^{-1})**	**Daytime hours at rest**
**COOMALLO CREEK**	2017	CB02	F	1	2270	13-Nov-17	1-Dec-17	15	58.53 ± 1.24	2.01 ± 0.26	3.20 ± 0.34	3.33 ± 0.19	23.01 ± 2.25	4.82 ± 0.38	5.06 ± 0.20
	2017	CB04	F	1	2313	15-Nov-17	2-Dec-17	14	62.86 ± 0.97	0.86 ± 0.13	3.71 ± 0.26	3.29 ± 0.22	33.14 ± 2.61	4.45 ± 0.21	6.23 ± 0.37
	2018	CB06	F	1	2435	15-Nov-18	20-Dec-18	27	64.55 ± 0.72	0.92 ± 0.12	4.87 ± 0.33	2.34 ± 0.09	28.80 ± 1.34	3.80 ± 0.19	6.04 ± 0.24
**BORDEN**	2017	CB09	M	2	2315	11-Dec-17	17-Dec-17	4	66.75 ± 1.31	0.56 ± 0.10	5.97 ± 0.20	2.25 ± 0.25	27.27 ± 2.62	3.69 ± 0.42	6.73 ± 0.38
	2017	CB10	M	2	2318	13-Dec-17	12-Jan-18	29	66.29 ± 0.69	1.52 ± 0.12	3.54 ± 0.29	2.74 ± 0.13	24.73 ± 1.77	3.35 ± 0.18	6.95 ± 0.24
	2018	CB11	M	1	2436	10-Dec-18	13-Dec-18	2	64 ± 4.0	1.84 ± 0.10	3.99 ± 0.15	2.5 ± 0.5	32.9 ± 10.47	5.99 ± 2.34	4.01 ± 1.14
	2018	CB13	M	1	2440	11-Dec-18	23-Dec-18	8	66.75 ± 0.90	1.22 ± 0.17	3.91 ± 0.51	2.13 ± 0.13	29.61 ± 3.31	3.81 ± 0.37	6.79 ± 0.40
	2018	CB14	M	1	2442	12-Dec-18	8-Jan-19	24	59.46 ± 1.41	1.33 ± 0.10	3.52 ± 0.22	2.04 ± 0.04	28.91 ± 1.62	3.88 ± 0.26	6.90 ± 0.35

Table S2: Response variables used in LMMs describing the effects of external factors on daily activity and foraging metrics.

LMM Response variable	Unit	Description
ODBA	ms^{-1}	Mean ODBA (proxy for energy expenditure) of all daytime GPS locations.
Distance travelled	km	Sum of all distances between consecutive daytime GPS locations.
Time at rest	hrs	Sum of the duration between subsequent daytime GPS locations following each location classified as resting.
Time spent foraging on canola	hrs	Sum of the duration between subsequent daytime GPS locations following each location classified as canola foraging.
Energy expended foraging on canola	ms^{-1}	Mean ODBA (proxy for energy expenditure) of all daytime locations classified as foraging on canola.
Time spent foraging in native vegetation	hrs	Sum of the duration between subsequent daytime GPS locations following each location classified as foraging in native vegetation.
Energy expended foraging on native vegetation	ms^{-1}	Mean ODBA (proxy for energy expenditure) of all daytime locations classified as foraging in native vegetation.

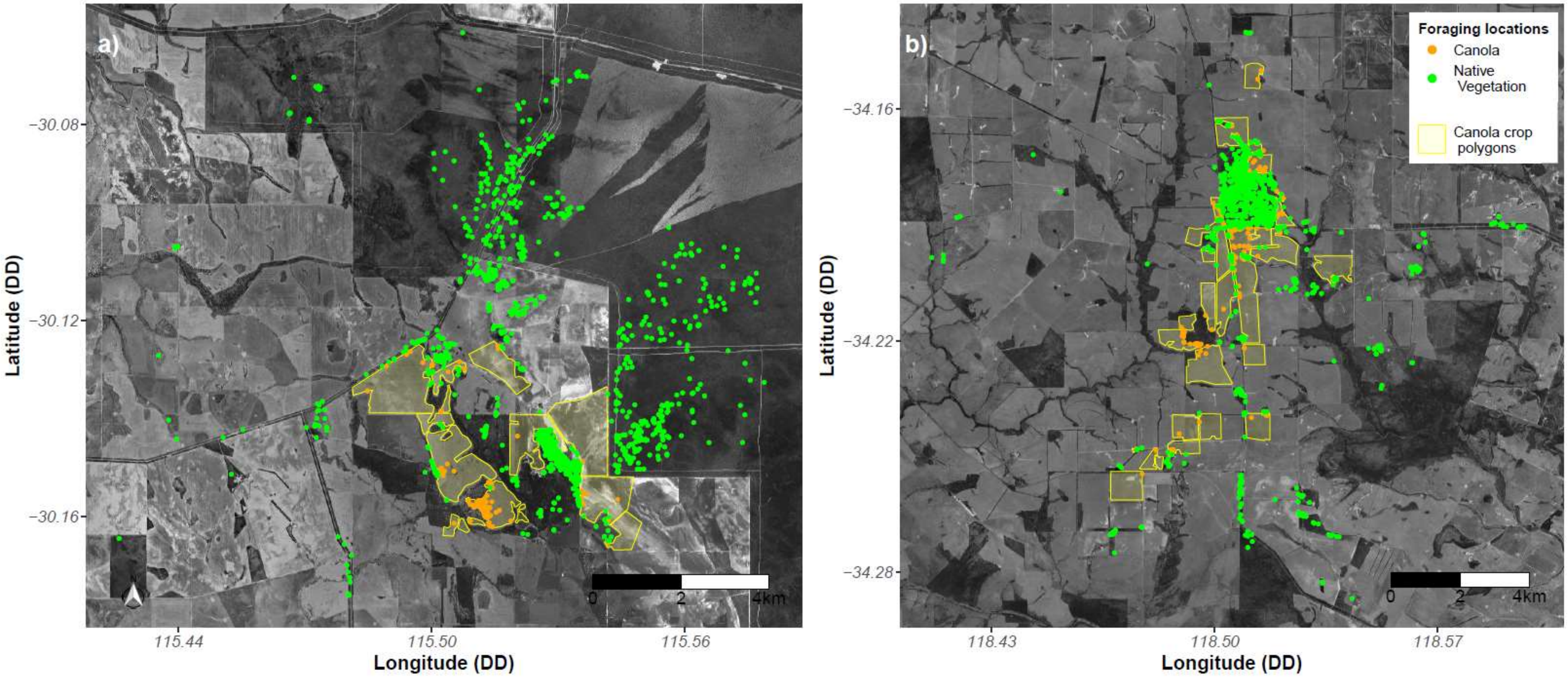

Figure S1: GPS locations where cockatoos were foraging on either canola (orange dots) or native vegetation (green dots) at a) Coomallo Creek, and b) Borden. Yellow polygons indicate canola crops (only canola crop polygons intersecting with foraging locations are depicted).

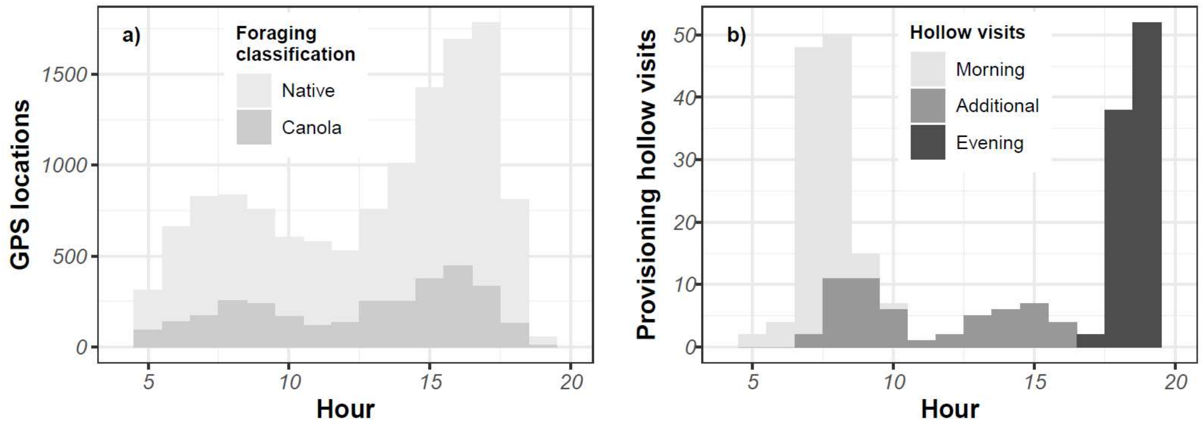

Figure S2: Histograms for cockatoo behaviour, showing timing of a) GPS locations classified as foraging on either native vegetation or canola, and b) provisioning nest hollow visits. All days included morning and evening activity. Data within columns are overlaid (not stacked).

Table S3: LMMs for factors affecting daily energetic and movement metrics (ODBA, distance travelled and time at rest). Proportion of foraging time spent in canola, site, maximum temperature, number of PHVs, nestling age and number of nestlings were included as fixed effects in all initial models, with non-significant effects removed in a stepwise fashion.

			95% Confidence Intervals			
**Response Variable**	**Fixed Effects**	**Estimated factor level mean or coefficient ± SE**	**Lower**	**Upper**	**χ^2**	**P-value**
Mean daytime ODBA (m/s^{-1})	(Intercept)				68.71	2.20x10^{-16} ***
	Proportion of foraging in canola	-3.08 ± 0.73	-4.52	-1.64	17.87	2.36x10^{-5} ***
	Maximum temperature (°C)	-0.08 ± 0.02	-0.12	-0.04	16.72	4.33x10^{-5} ***
	Number of nestlings: 1	4.30 ± 0.32	3.52	5.09	2.18	0.14
	Number of nestlings: 2	3.35 ± 0.56	1.98	4.72		
Distance (km)	(Intercept)				0.24	0.63
	Proportion of foraging in canola	-26.1 ± 5.07	-36.20	-16.20	26.5	1.71x10^{-6} ***
	Maximum temperature (°C)	-0.65 ± 0.15	-0.90	-0.36	19.17	2.73x10^{-5} ***
	Nestling Age (days)	0.21 ± 0.08	0.05	0.37	6.96	0.01 *
	Number of nestlings: 1	29.0 ± 0.91	26.70	31.40	4.76	0.16
	Number of nestlings: 2	24.3 ± 1.88	19.40	29.10		
	Provisioning nest visits	1.71 ± 1.09	-0.44	3.87	2.48	0.12
	Daylength (hours)	6.12 ± 6.71	-7.18	19.40	0.83	0.37
	Site: Borden	26.9 ± 2.33	20.90	32.90	0.08	0.93
	Site: Coomallo Creek	26.4 ± 3.05	18.60	34.20		
Time at Rest (hrs)	(Intercept)				0.43	0.51
	Proportion of foraging in canola	1.97 ± 0.83	0.33	3.61	5.68	0.01 *
	Maximum temperature (°C)	0.11 ± 0.02	0.07	0.16	25.41	4.62x10^{-7} ***
	Nestling Age (days)	0.05 ± 0.01	0.03	0.08	13.18	1.23x10^{-4} ***
	Daylength (hours)	-0.75 ± 1.15	-3.02	1.52	0.43	0.51
	Site: Borden	7.12 ± 0.54	5.85	8.39	3.35	0.07
	Site: Coomallo Creek	5.28 ± 0.62	3.76	6.81		

Table S4: LMMs for factors affecting daily allocation of time and energy to canola and native vegetation foraging. Provisioning hollow visits, site, maximum temperature, daylength, nestling age and number of nestlings were included as fixed effects in all initial models, with non-significant effects removed in a stepwise fashion. No significant effects were found in the model testing canola foraging energy expenditure (mean ODBA (m/s^{-1})); no model for this response variable is displayed.

Response Variable	Fixed Effects	Estimated factor level mean or coefficient ± SE	95% Confidence Intervals Lower	Upper	χ^2	P-value
Canola foraging time (hrs)	(Intercept)				53.32	2.82x10^{-13} ***
	Provisioning hollow visits	0.197 ± 0.09	0.01	0.38	16.79	0.03*
	Nestling Age (days)	-0.013 ± 0.01	-0.03	0.00	0.24	0.06
	Number of nestlings: 1	1.25 ± 0.17	0.84	1.65	0.66	0.95
	Number of nestlings: 2	1.26 ± 0.25	0.66	1.86		
Native foraging time (hrs)	(Intercept)				6.19	1.28x10^{-2} *
	Provisioning hollow visits	-0.71 ± 0.19	-1.09	-0.33	13.51	2.37x10^{-4} ***
	Temperature (°C)	-0.16 ± 0.02	-0.21	-0.11	39.92	2.65x10^{-10} ***
	Daylength (hours)	3.66 ± 1.88	1.33	6.00	9.65	1.89x10^{-3} **
	Site: Coomallo Creek	5.97 ± 0.45	5.11	7.30	23.64	1.16x10^{-6} ***
	Site: Borden	2.95 ± 0.4	1.50	3.39		
	Nestling Age (days)	-0.03 ± 0.01	-0.06	0.0001	9.65	0.048*
	Number of nestlings: 1	3.96 ± 0.15	3.59	4.33	3.62	0.057
	Number of nestlings: 2	4.69 ± 0.33	3.87	5.51		
Native foraging effort (mean ODBA (m/s^{-1}))	(Intercept)				68.3	2.00x10^{-16} ***
	Temperature (°C)	-0.03 ± 0.01	-0.05	-0.005	5.68	0.02*
	Nestling Age (days)	-0.02 ± 0.01	-0.03	-0.003	6.47	0.01*
	Number of nestlings: 1	3.67 ± 0.06	3.51	3.82	5.29	0.02*
	Number of nestlings: 2	3.31 ± 0.13	2.99	3.63		

Table S4: Energetic content of seed samples identified as foraging species. Individual seed/nut mass for banksia and hakea spp. presented as a range; seed mass for canola, pine and wild radish presented as a mean (n=30).

Species	Sample (N)	Individual seed/ nut mass (g)	Mean energy content (kJ/g)
Banksia (*B. armata, B. candolleana, B. fraseri, B. gardneri, B. sphaerocarpa and B. tenuis*)	8	0.01 to 0.12	21.42 ± 0.46
Hakea (*H. auriculata, H. laurina, H. smilacifolia and H. prostrata*)	4	0.01 to 0.10	20.79 ± 0.26
Brassica napus (Canola)	9	0.0035 ± 0.0007	28.7 ± 0.47
Pinus pinaster	2	0.001 ± 0.0003	26.3 ± 0.49
Raphanus raphanistrum (Wild radish)	1	0.003 ± 0001	27.5
